# CD44 Expression Intensity Marks Colorectal Cancer Cell Subpopulations with Different Extracellular Vesicle Release Capacity

**DOI:** 10.3390/ijms23042180

**Published:** 2022-02-16

**Authors:** Andrea Kelemen, Idan Carmi, Iván Seress, Péter Lőrincz, Tamás Tölgyes, Kristóf Dede, Attila Bursics, Edit I. Buzás, Zoltán Wiener

**Affiliations:** 1 Department of Genetics, Cell and Immunobiology, Semmelweis University, H-1089 Budapest, Hungary; kelemen.andrea1@med.semmelweis-univ.hu (A.K.); idan.carmi@gmail.com (I.C.); ivan.seress@gmail.com (I.S.); edit.buzas@gmail.com (E.I.B.); 2 Department of Anatomy, Cell and Developmental Biology, Eötvös Loránd University of Sciences, H-1117 Budapest, Hungary; concrete05@gmail.com; 3 Uzsoki Hospital, H-1145 Budapest, Hungary; tolgyestamas5@gmail.com (T.T.); dede.kristof@gmail.com (K.D.); abursics@gmail.com (A.B.); 4 MTA-SE Immune-Proteogenomics Extracellular Vesicle Research Group, Semmelweis University, H-1089 Budapest, Hungary; 5 HCEMM-SE Extracellular Vesicle Research Group, H-1089 Budapest, Hungary

**Keywords:** exosome, cancer stem cell, organoid, cancer-associated fibroblast, CD44, CD133, PTK7

## Abstract

Extracellular vesicles (EV) are released by virtually all cells and they transport biologically important molecules from the release site to target cells. Colorectal cancer (CRC) is a leading cause of cancer-related death cases, thus, it represents a major health issue. Although the EV cargo may reflect the molecular composition of the releasing cells and thus, EVs may hold a great promise for tumor diagnostics, the impact of intratumoral heterogeneity on the intensity of EV release is still largely unknown. By using CRC patient-derived organoids that maintain the cellular and molecular heterogeneity of the original epithelial tumor tissue, we proved that CD44^high^ cells produce more organoids with a higher proliferation intensity, as compared to CD44^low^ cells. Interestingly, we detected an increased EV release by CD44^high^ CRC cells. In addition, we found that the miRNA cargos of CD44^high^ and CD44^low^ cell derived EVs largely overlapped and only four miRNAs were specific for one of the above subpopulations. We observed that EVs released by CD44^high^ cells induced the proliferation and activation of colon fibroblasts more strongly than CD44^low^ cells. However, this effect was due to the higher EV number rather than to the miRNA cargo of EVs. Collectively, we identified CRC subpopulations with different EV releasing capabilities and we proved that CRC cell-released EVs have a miRNA-independent effect on fibroblast proliferation and activation.

## 1. Introduction

Extracellular vesicles (EV) are membrane-enclosed structures released by virtually all cell types. EVs form a heterogeneous population both by size and cellular origin. In contrast to small EVs (exosomes) derived from the multivesicular bodies (MVBs) of the endosomal–lysosomal compartment of cells, larger EVs including microvesicles (MVs) are directly shed from the plasma membrane [1,2]. Since most methods separate EVs based on their size and not on their cellular origin, EVs are often categorized as small (sEV), medium (mEV), and large (lEVs) EVs [3]. Importantly, EVs carry biologically active molecules, such as miRNAs, lipids, and proteins from the releasing to the target cells, thus, providing a special way of intercellular signal transmission. Since EVs transport their cargo in a protected way in the tissues and body fluids, and molecules specific for the releasing tumor cells are thought to be represented at a high concentration in EVs, they provide a promising tool for early cancer diagnostics. Although EVs are thought to be involved in intercellular communication, how the presence of different tumor cell subpopulations affects the release, cargo, and functions of EVs is not yet known.

Colorectal cancer (CRC) is among the most frequent cancer types in developed countries. In the majority of CRC patients, mutation in the *APC* gene is an initializing genetic change, leading to the continuous and ligand-independent activation of the Wnt pathway, uncontrolled expression of Wnt target genes, and the proliferation of intestinal epithelial cells. This adenoma stage can progress to invasive carcinomas with the accumulation of other mutations [4]. Interestingly, tumor cells within the same tumor show a large degree of genetic, molecular, and as a consequence, phenotypic variation, leading to intra-tumoral cellular heterogeneity. For example, CD133, a transmembrane glycoprotein, is associated with tumor-initiating cells (CIC) of malignant behavior [5] and protein-tyrosine pseudokinase 7 (PTK7), a component of the Wnt signaling pathway, is expressed in stem cells of the human colon [6] and marks a CRC cell population with motility and metastasis [7]. In addition, PTK7+ CRC cells have a high Wnt and colony-forming activity in CRC cell line models [8], suggesting that PTK7 marks an aggressive CRC cell population.

CD44, involved in cell adherence and signaling, exerts pleiotropic effects on proliferation, migration, survival, and epithelial to mesenchymal transition (EMT), and in addition to CD133 and PTK7, CRC cells show a differential expression level of this molecule. Several studies have analyzed the features of CD44^high^ and CD133^high^ CRC cell populations and most of them suggested that CRC stem cells also express these molecules [9,10]. Although the identity and function of CRC stem cells are subjects of intensive discussion, high CD44 or CD133 expression clearly characterizes CRC cell subpopulations with aggressive properties [5,11,12,13]. Thus, CRC contains tumor cell populations with different molecular profiles. Understanding of the critical role of this intra-tumoral cellular heterogeneity in drug resistance, relapse and metastasis has just started to emerge.

Cancer-associated fibroblasts (CAFs) represent one of the most abundant stromal cell types in the tumor tissue of CRC. They are a group of activated fibroblasts with a significant role in tumor microenvironment formation and tumor-promoting functions [14]. Fibroblast activation is associated with an increased expression in a characteristic group of genes including *CDH2*, *ENC1*, *TNFSF4*, *ST6GALNAC5*, *SEMA5A*, *SLC7A2*, and *TGFB2* [15]. The accumulation of activated fibroblasts in the CRC tissue is associated with a significantly lower patient survival and a higher relapse frequency [16]. These fibroblasts secrete e.g., IL-11 that triggers GP130/STAT3 signaling in tumor cells, leading to metastasis initiation [17]. CAFs also produce factors that induce the stem cell features of surrounding cancer cells [18]. In our previous study, we also proved that fibroblast-derived EVs carried amphiregulin and critically they induced CRC cell proliferation [19]. Thus, activated fibroblasts play an important role in shaping the microenvironment for cancer cells.

Patient-derived organoids maintain the cellular heterogeneity of the in vivo tumors of epithelial origin, thus, this technology represents one of the most state-of-the-art methods to study human cancers [20,21]. Importantly, they are widely used in developmental studies, in uncovering the mechanisms of human tumors, and in patient-specific drug screening. By using CRC patient-derived organoids, we addressed the role of cellular heterogeneity in the release and function of CRC cell-derived EVs. We set out to identify molecules that mark CRC cell subpopulations with different EV release capabilities, focusing on markers of aggressive CRC cells. Interestingly, we found that unlike PTK7^high^ and CD133^high^ cells, CD44^high^ CRC cells produced more EVs compared to CD44^low^ cells. However, we did not find a large difference in their miRNA cargo. EVs derived from both subpopulations could activate colon fibroblasts and this effect depended on the number of EVs, but not on their cargo. Thus, CD44^high^ CRC cells may have a higher activating effect on fibroblasts by their higher EV release intensity as compared to CD44^low^ cells.

## 2. Results

### 2.1. CD44 and CD133, but Not PTK7 Mark CRC Organoid Cell Populations with a High Proliferative Potential

**Figure 1:**
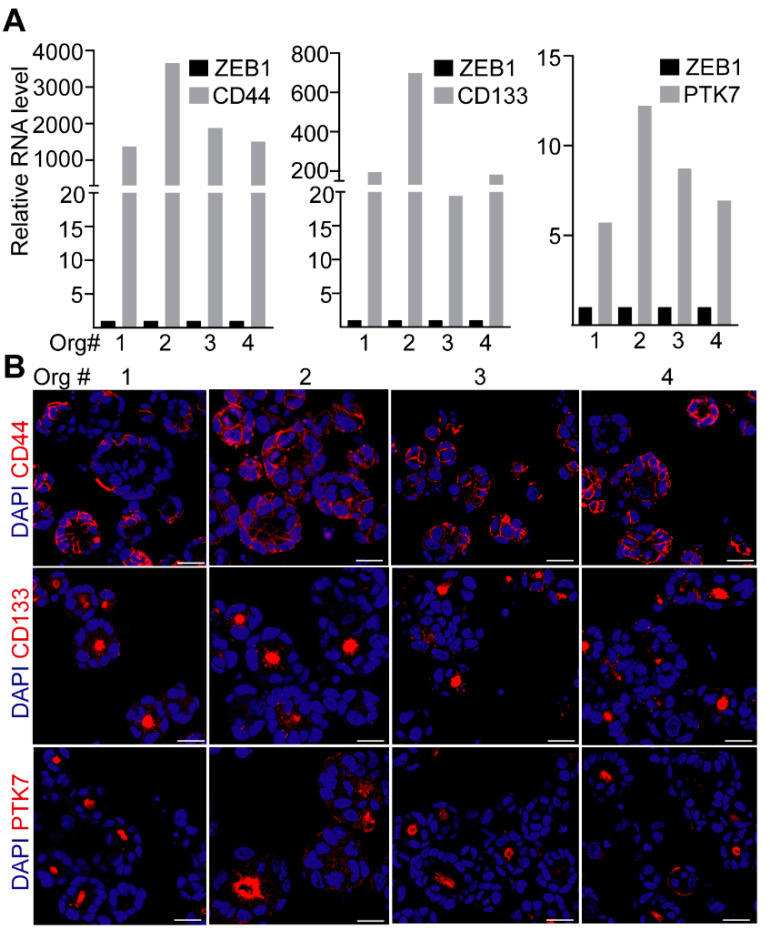
Heterogeneous expression of CD44, CD133, and PTK7 in CRC organoids. (**A**) The RNA level for the indicated genes in organoids #1,2,3 and #4. RNA levels were normalized to the *HPRT1* housekeeping gene and then the normalized *ZEB1* level was taken as 1. (**B**) Whole-mount immunostaining for CD44, CD133, and PTK7. Scale bars: 50 µm.

Many molecules have already been published that mark aggressive cell populations in CRC, such as CD44, CD133, and PTK7. To study the intra-tumoral heterogeneity for these molecules, we used CRC patient-derived organoids. We detected the RNAs of these markers in all our organoid lines. Although the RNA levels of *CD44*, *CD133* and *PTK7* varied among patient-derived organoids, we consequently detected a markedly higher level for these markers in all organoids compared to the mesenchymal marker *ZEB1* that we used as a control (Figure 1A). Immunostaining showed that whereas CD133 and PTK7 were localized to the apical surface of some CRC cells, we did not find such an asymmetrical distribution of CD44 (Figure 1B). In addition, we observed cellular heterogeneity for the expression of CD44, CD133, and PTK7 within and among the organoids from the same patient (Figure 1B). Of note, all three molecules were detected in all four organoid lines used in our studies (Figure 1B).

**Figure 2:**
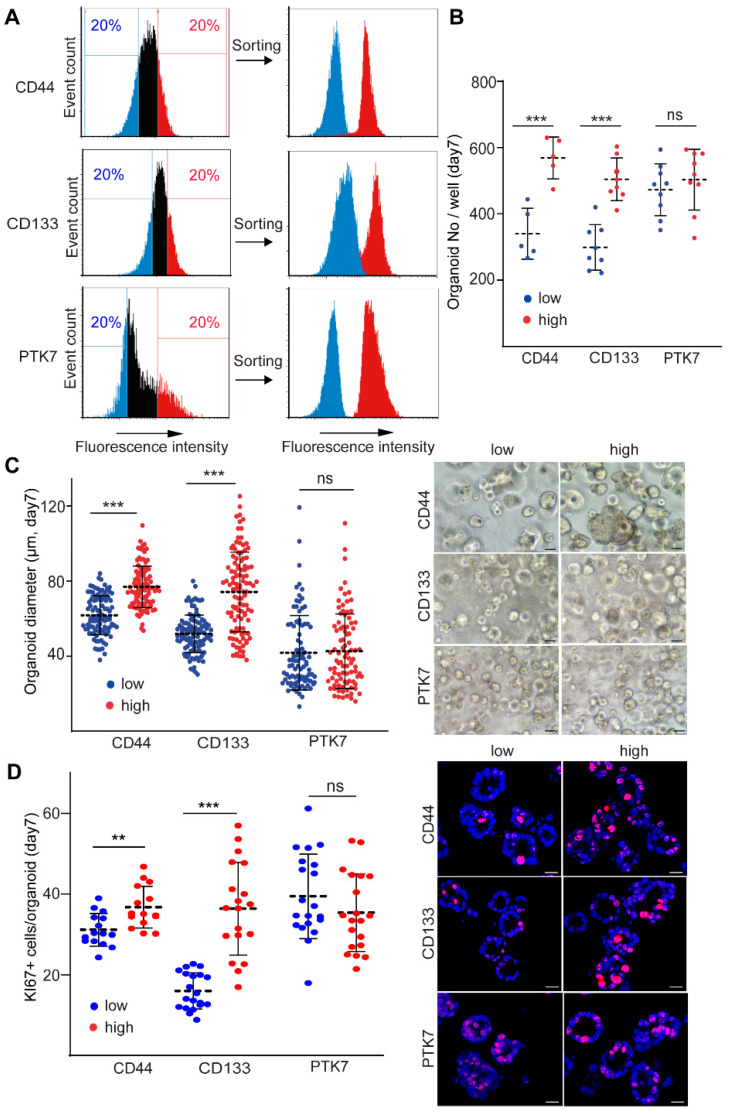
CD44^high^ and CD133^high^ cell-derived organoids have more proliferating cells as compared to CD44^low^ and CD133^low^ organoids. (**A**) Sorting strategy and the level of CD44, CD133, and PTK7 in the sorted populations. (**B**) Number of organoids produced from cells sorted for low and high levels of the respective markers, counted on day7 (1–2 sorting experiments from organoid lines #1–#4). (**C**) The diameter of the organoids 7 days after seeding the sorted cells into Matrigel. (**D**) The percentage of KI67+ proliferating cells in the organoids (confocal microscopic images). For C and D, quantification (left panel) and representative images (right panel) are shown (two sorting experiments from four organoid lines). Scale bars: 25 µm (**C**,**D**). Mann–Whitney U test was used (**B**–**D**). ** *p* < 0.01, and *** *p* < 0.005.

To analyze cell subpopulations marked by CD44, CD133, and PTK7, we sorted CRC organoid cells with the highest and lowest level of these molecules (Figure 2A). As expected, these cell populations showed different levels of the markers directly after sorting (Figure 2A). Whereas we found no difference in the colony-forming ability when comparing PTK7^high^ and PTK7^low^ cells, we observed an elevated number of organoids derived from CD44^high^ and CD133^high^ cells as compared to CD44^low^ and CD133^low^ cells, respectively (Figure 2B). Moreover, we detected an increase in the diameter and percentage of KI67+ proliferating cells in the organoids grown from CD44^high^ and CD133^high^, but not from PTK7^high^ CRC cells when compared to organoids from cells with low levels of the respective marker (Figure 2C,D). Importantly, we found no change in the proportion of active caspase-3+ apoptotic cells in any subpopulations (Figure S1A). Collectively, these results suggest that CD44 and CD133, but not PTK7 mark CRC cells of high proliferative potential.

### 2.2. CD44^high^ CRC Cell-Derived Organoids Release More EVs Than CD44^low^ Organoids

**Figure 3:**
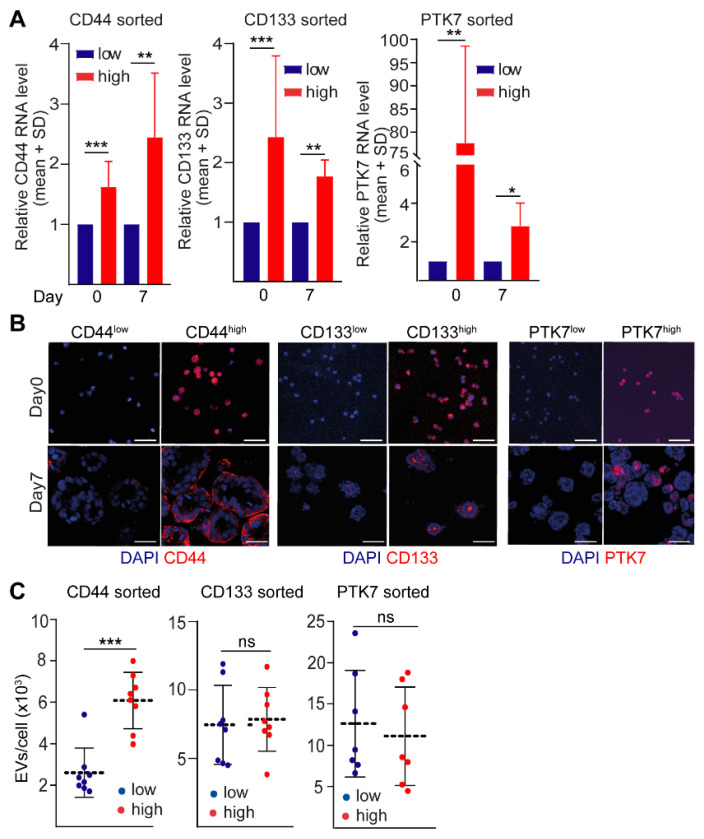
CD44^high^ cells release more EVs compared to CD44^low^ CRC cells. (**A**) Relative RNA levels for the indicated genes directly after sorting for CD44, CD133, or PTK7 (d0) or 7 days after organoid formation. Data were normalized to the housekeeping control and the expression level of the CD44^low^, CD133^low^ or PTK7^low^ population was taken as 1 (RT–qPCR, *n* = 4 from four organoid lines). (**B**) Immunostaining for CD44, CD133, and PTK7 directly after sorting (d0) or 7 days after culturing (confocal microscopic analysis, organoid #3). (**C**) EV concentration in the supernatants of organoids 7 days after sorting (1–2 parallels from four organoid lines, normalized to cell number). Scale bars: 50 µm (**B**). Paired *t*-test (**A**) and Mann–Whitney U test were used (**C**). * *p* < 0.05, ** *p* < 0.01, and *** *p* < 0.005.

To determine how cellular heterogeneity impacts the intensity of EV release, we cultured cells sorted according to their level of CD44, CD133 or PTK7 in 3D conditions. Importantly, the sorted cell-derived organoids maintained the high or low expression pattern for these molecules at day 7, determined by RT–qPCR (Figure 3A) and immunocytochemistry (Figure 3B). Thus, we decided to quantify EVs from organoids on day 7 after sorting. We confirmed the presence of EVs in the conditioned media by using anti-CD63 or anti-CD81-coated beads and flow cytometry (Figure S1B). Importantly, CD63 and CD81 are characteristic markers of EVs. In addition, nanoparticle tracking analysis (NTA) demonstrated the presence of EVs in organoid cultures, but we could hardly detect any EVs in the negative control samples without cells (Figure S1C, data from CD44^low^ and CD44^high^ organoids are shown). Of note, these NTA measurements were carried out from conditioned media without EV isolation, thus, they are in agreement with our previous publication showing that the smaller EVs are released preferentially from the 3D matrix (Matrigel), into the supernatant [22]. In addition, we detected EVs in organoid culture media after ultracentrifugation, a widely accepted method for EV isolation and concentration, with transmission electron microscopy (TEM) (Figure S1D) and NTA (Figure S1E). Furthermore, capillary-based immunoblot analysis showed that these EV preparations contained the sEV marker TSG101 [2] (Figure S1F). Thus, both conditioned media and the ultracentrifuged pellet of organoid cultures contained EVs.

Organoids derived from PTK7^high^ or CD133^high^ cells did not differ in their EV release as compared to PTK7^low^ or CD133^low^ cells as measured by NTA (Figure 3C). In contrast, we found a higher EV secretion from CD44^high^ organoids (Figure 3C). Of note, collagen I accumulates during CRC progression and it demonstrates a major change of the extracellular matrix. Interestingly, CD44^high^ cell-derived organoids showed an enhanced EV secretion also in the presence of collagen I and in the mixture of collagen I and Matrigel (1:1 ratio) compared to CD44^low^ cells (Figure S2A,B). Since we normalized all EV release data to cell numbers, this approach ensured that EV secretion differences did not reflect the varying cell proliferation under different conditions. Thus, our data indicate that high CD44 level marks a CRC cell population with high EV release.

### 2.3. CD44^high^ and CD44^low^ CRC Cell-Derived EV Cargos Differ Only in a Few miRNAs

**Figure 4:**
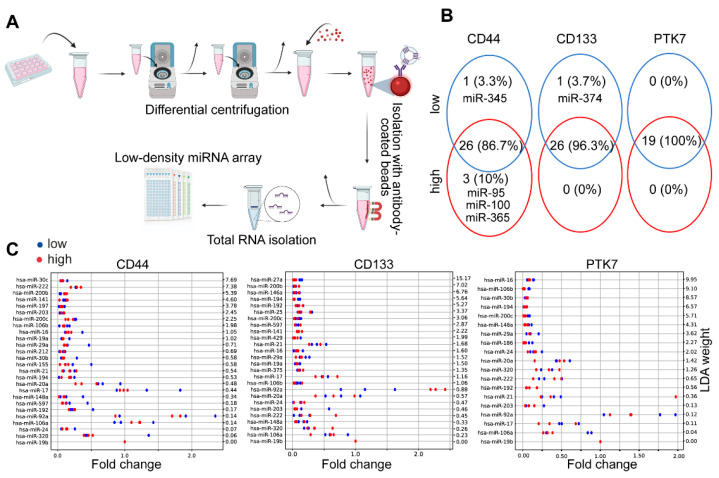
No large difference in the EV miRNA cargo between CD44^high^ and CD44^low^, CD133^high^ and CD133^low^, PTK7^high^ and PTK7^low^ cells. (**A**) The workflow of the EV isolation and miRNA cargo analysis. (**B**) Venn diagram of the overlapping and non-overlapping miRNAs, detected on day7 after organoid initiation from the sorted cells. The number and the percentage of the miRNAs are represented. (**C**) LDA analysis of the normalized miRNA levels. Note that miRNAs present in all samples of the corresponding experimental groups were selected.

As a next step, we compared the cargo of EVs from CD44^high^ and CD44^low^, CD133^high^ and CD133^low^ or PTK7^high^ and PTK7^low^ cell-derived organoids. Since miRNAs transported by EVs are thought to be of outstanding importance in cell–cell communication, we focused on these molecules. To obtain a pure EV preparation, we captured EVs from organoid conditioned media by anti-CD63 and anti-CD81-coated beads, a method producing the lowest unspecific miRNA background according to our previous data [23]. We then screened EV miRNAs with low-density miRNA arrays (Figure 4A). As controls, conditioned media from cell-free Matrigel samples were collected, the detected miRNAs were considered as background and they were excluded from further analysis. Of note, we only focused on miRNAs that were present in all the biological parallel samples of a minimum of one experimental condition. By applying these criteria, we detected 26, 26, and 19 miRNAs out of the 377 analyzed miRNAs when comparing organoids with different CD44, CD133, or PTK7 levels, respectively (Figure 4B and Table S4). We found only one miRNA differentially expressed by CD133^high^ and CD133^low^ organoid-derived EVs and no miRNAs were found to be specific for PTK7^high^ and PTK7^low^ CRC cell-derived EVs. On the other hand, our bioinformatical analysis indicated that miR-95, miR-100, and miR-365 were specific for the CD44^high^ and miR-345 for the CD44^low^ organoid-derived EVs (Figure 4B). As the next step, we normalized EV cargo miRNAs to miR-19b levels. We used these normalized fold change values for linear discriminant analysis (LDA) to find miRNAs with differing levels between the experimental groups. Of note, miR-19b had the most stable level across our samples. When focusing only on miRNAs that were present in both the CD44^high^ and the CD44^low^ organoid-derived EVs, this bioinformatical method showed a higher level of miR-20a in CD44^low^ cell-derived EVs as compared to EVs released by CD44^high^ cells (Figure 4C). Similarly, we found some differences in the levels of miR-27a, miR-92a, and miR-203 between EVs released by CD133^high^ and CD133^low^ organoids. However, EVs derived from different PTK7 subpopulations did not show any difference in their miRNA cargo patterns (Figure 4C). Thus, we concluded that EVs released by CRC subpopulations have only a marginal difference in their miRNA cargo.

### 2.4. Dose-Dependent Effect of CD44^high^ and CD44^low^ CRC Organoid-Derived EVs on Fibroblasts

**Figure 5:**
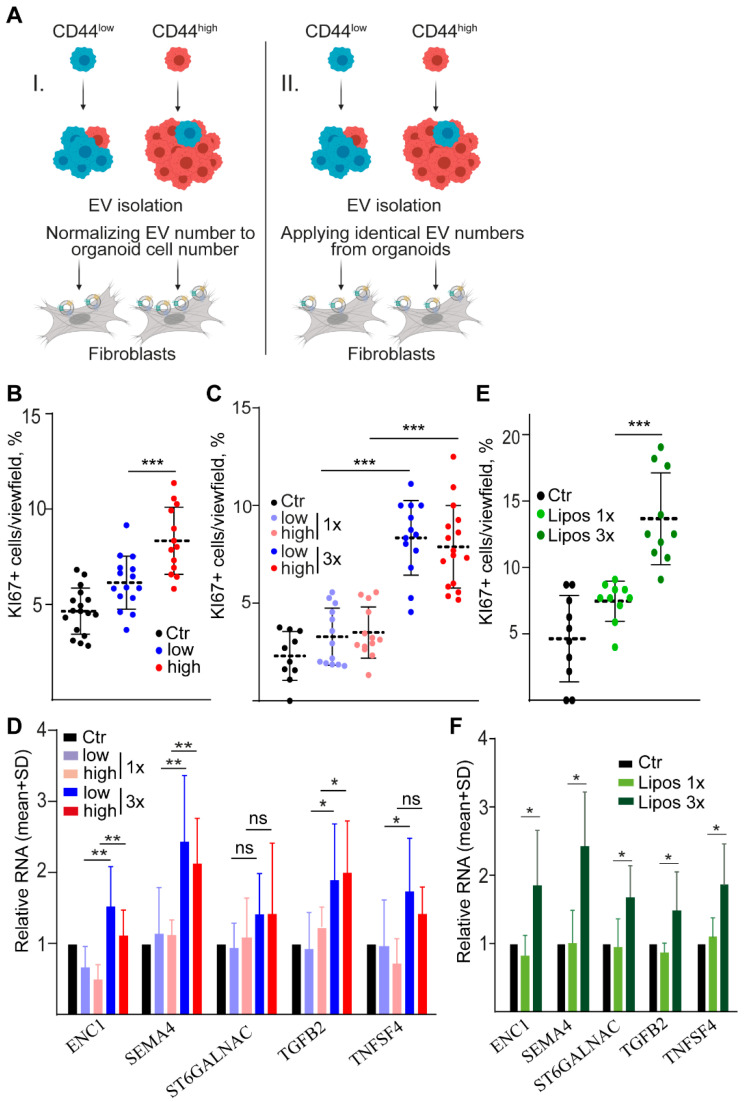
EV effect on fibroblasts depends on the EV dose, but not on the miRNA cargo. (**A**) Schematic representation of the experimental setups. Note that fibroblasts were treated with EVs either from identical cell numbers (left panel, setup I) or with identical EV numbers derived from different CRC subpopulations (right panel, setup II). (**B**) The percentage of KI67+ colon fibroblasts when applying EVs from 10^6^ cells. EVs were collected from the supernatants of CD44^high^ or CD44^low^ cell-derived organoids on day 7 and fibroblasts were treated for 48h (quantification of confocal microscopic images, experimental setup I). Note that control samples received no EVs. (**C**) The percentage of proliferating fibroblasts when applying increasing numbers of CD44^high^ or CD44^low^ organoid-derived EVs (experimental setup II). (**D**) RNA levels for the indicated genes in fibroblasts treated with CD44^high^ or CD44^low^ cell-derived EVs at different concentrations (setup II). Data were normalized to the housekeeping gene and then compared to the control (Ctr) samples that received no EVs (RT–qPCR, *n* = 4 from four organoid lines). (**E**) The percentage of KI67+ fibroblasts when treated with different numbers of liposomes (quantification of confocal microscopic images from 3 experiments). (**F**) Relative RNA levels of the indicated genes when fibroblasts were treated with different amounts of liposomes (RT–qPCR, *n* = 4 from independent experiments). EVs from four organoid lines were used for B–D. Kruskal–Wallis and Dunn tests (**B**,**C**,**E**) or paired *t*-test (**D**,**F**) were used. EV and liposome numbers were 2 × 10^5^/200 µL medium (1×) and 6 × 10^5^/200 µL (3×) (**C**–**F**). * *p* < 0.05, ** *p* < 0.01, and *** *p* < 0.005.

To study the functional importance of the differential miRNA cargo and secretion intensity of EVs from CD44^high^ and CD44^low^ CRC cells, we tested the effects of EVs on fibroblasts, a cell type that plays a critical role in shaping the microenvironment for CRC cells. Interestingly, when using EV amounts normalized to cell number (see experimental setup I on Figure 5A), EVs secreted by CD44^high^ organoids induced a higher proportion of KI67+ proliferating colon fibroblasts compared to CD44^low^ cell-derived EVs (Figure 5B). This difference was not observed when testing EVs from identical numbers of CD133^high^ and CD133^low^ or PTK7^high^ and PTK7^low^ cells (Figure S3A,B). To decide whether this effect depended on the differential EV release by CD44^high^ and CD44^low^ cells or on the differential miRNA cargo, we repeated the experiments with an increasing amount of EVs isolated from both CRC cell subpopulations (see experimental setup II on Figure 5A). Of note, we observed that the percentage of KI67+ fibroblasts depended on the EV dose, but it was independent of whether they had been isolated from CD44^high^ or CD44^low^ organoids (Figure 5C). In addition, we found no difference in the uptake intensity of CD44^high^ and CD44^low^ cell-derived EVs by fibroblasts using a previously validated method [24] (Figure S3C,D). Importantly, EVs were collected from sorted cell-derived organoids that had been pre-treated with a membrane labelling dye in these experiments. These results indicate that it is not the differential miRNA cargo, but the number of EVs that is the critical factor. Furthermore, the expression of activation markers in colon fibroblasts [15] depended on the number of EVs, and again, we could not find a difference between CD44^low^ and CD44^high^ cell-derived EVs (Figure 5D). Interestingly, the increasing amounts of artificially produced liposomes had a similar, dose–dependent effect on both the proliferation rate and the expression of activation markers in fibroblasts (Figure 5E,F and Figure S3E), suggesting that the common miRNA cargo of CD44^low^ and CD44^high^ cell-derived EVs is not critical either. Collectively, these results indicate that: (i) fibroblast activation is induced by EVs or liposomes in a dose–dependent manner possibly via their lipid components; (ii) the higher EV secretion by CD44^high^ CRC cells, and not the differential miRNA cargo is important in the differential effects of EVs derived from CD44^high^ and CD44^low^ cells.

## 3. Discussion

Here we set out to identify CRC cell subpopulations with different EV release capabilities. We focused on molecules (PTK7, CD133, CD44) that are markers of CRC cell subpopulations with aggressive behavior [5,7,11]. Out of these markers, we found that a high level of CD44 marked a CRC cell population in patient-derived organoids with a higher proliferative capacity and a higher EV release as compared to CD44^low^ cells. Interestingly, we found the differential presence of only four miRNAs when comparing the cargo of EVs derived from these two subpopulations. In addition, EVs stimulated the proliferation and induced the expression of activation markers of fibroblasts in a dose–dependent manner. We received a similar effect when using an increasing number of liposomes. Thus, CD44^high^ CRC cells exerted a stronger effect on fibroblast proliferation and activation by their enhanced EV release as compared to CD44^low^ cells, but this effect did not depend on the miRNA cargo of the EVs.

PTK7 has been shown to mark a cell population with high Wnt activity [8]. Interestingly, in contrast to other reports, we found no significant difference in organoid forming efficiency and cell proliferation when sorting PTK7^high^ and PTK7^low^ cells [8]. Since in previous publications CRC cell line-derived spheroids were used, which differ in several features from patient-derived organoids, this may explain the discrepancy and may suggest that PTK7 does not mark a cell population with stem cell features in all models and/or patients.

MicroRNAs (miRNAs) are widely studied non-coding RNAs that critically regulate gene expression. Several studies have detected miRNAs in body fluids. A large proportion of circulating miRNAs are concentrated in EVs [25] and may stimulate or inhibit the proliferation of target cells [26]. Interestingly, we detected 26 common miRNAs in EVs derived from either CD44^high^ or CD44^low^ CRC cells. A meta-analysis suggested that the majority of the detected miRNAs were present in the plasma samples of CRC patients compared to healthy controls as well [27]. Of note, we detected miR-95, miR-100, and miR-365 only in EVs derived from the CD44^high^ cell populations when sorting cells based on differential CD44 expression. miR-95 was shown to induce cell proliferation by suppressing sorting nexin1 (SNX1) expression in CRC [28]. miR-100 mediates cetuximab resistance by increasing the strength of Wnt signaling [29]. In addition, this miR inhibited cell proliferation in multiple systems, such as in CRC (by decreasing Lgr5 expression [30]) or in mammary tumor cells both in vitro and in vivo [31]. Similarly, miR-365 is frequently down-regulated in CRC. It inhibits cell cycle progression and induces apoptosis [32]. Interestingly, miR-365 has a contradictory role in different cancer types and some reports showed it having promoting, while others showed inhibiting effects on cell proliferation [33,34,35]. Importantly, miR-365 was also suggested as a biomarker in oral squamous cell carcinoma-derived EVs [36]. The only miR that we found to be specific for CD44^low^ CRC cell-derived EVs was miR-345 that is known to stimulate cell proliferation and invasion [37].

Interestingly, we found that an increasing number of EVs induced not only the expression of genes that are characteristic for activated fibroblasts, but also the proliferation of fibroblasts that represents one of the most abundant cell types in the CRC microenvironment. Of note, this effect was independent of whether EVs had been isolated from CD44^high^ or CD44^low^ cells. Although the miRNA cargo of EVs is generally considered an important player in intercellular communication, miRNAs differentially present in the two EV groups are unlikely to have a major effect in our model. In addition, we observed a similar dose–dependent effect on fibroblasts when applying liposomes, suggesting that the shared miRNA cargo of EVs between different CRC subpopulations does not play a dominant role either. Since liposomes used in this study were produced from phosphocholine and cholesterol without any further cargo, this raises the possibility that EVs and liposomes may exert their dose–dependent effect on fibroblasts by transferring lipid molecules. This notion is supported by the work from Sun et al. showing that cholesterol-induced many functions of prostate cancer cells, such as proliferation and migration [38]. Furthermore, cell-intrinsic and cell-extrinsic cues, reprogram cholesterol metabolism in multiple tumors and promote tumorigenesis [39]. In addition, lipid uptake can induce the proliferation of not only cancer cells, but of fibroblasts as well [40]. Further experiments are needed to clarify the mechanisms of how the lipid components of EVs and liposomes act in fibroblasts.

## 4. Material and Methods

### 4.1. Cell Cultures

Normal human colon fibroblasts (CCD-18Co, ATCC-1459) were cultured in DMEM containing 4500 g/L glucose (Gibco, Thermo Fisher Scientific, Waltham, MA, USA), 10% FBS (Biosera, Kansas, MO, USA), 1× penicillin/streptomycin (Gibco, Thermo Fisher, Waltham, MA, USA) and glutamine (Merck, Darmstadt, Germany). Cells were washed with phosphate-buffered saline (PBS) three times and cultured in serum-free medium when applying EVs. Cell number was counted in a Burker chamber. We only used cells with low (<p10) passage numbers after receiving them from the distributor. Cell cultures were regularly tested for Mycoplasma contamination with Hoechst staining and they were negative in our studies.

### 4.2. CRC Organoid Cultures

The Medical Research Council of Hungary (ETT-TUKEB, No 51323-4/2015/EKU) approved the experiments with human samples and informed consent was obtained from the patients. We used the CRC organoid lines established and characterized previously in our research group [22]. Organoids were cultured in CRC medium containing advanced DMEM/F12 (Gibco), 10 mM HEPES (Merck), penicillin/streptomycin (Gibco), glutamine (Gibco), B27 supplement (Gibco), 1 mM N-Acetyl-Cysteine (Merck), 10 mM Nicotinamide (Merck), 50 ng/mL EGF (Peprotech, London, UK), 10 uM SB202190-Monohydrochloride (Merck) and 500 nM A83-01 (Merck). The Rho kinase inhibitor Y27632 (Merck) was also added for 3 days after passaging to avoid anoikis. Organoids were removed from Matrigel every 5–6 days mechanically, centrifuged at 700× *g* for 5 min, washed with phosphate-buffered saline (PBS), and digested with TrypLE (Thermo Fisher, Waltham, MA, USA) until organoids were dissociated into cell clusters. Samples were then washed with advanced DMEM/F12 medium and embedded into Matrigel again in a 1:3 ratio. Organoids #1–4 have already been characterized and clinical data of the patients have been published [22,24].

### 4.3. Nanoparticle Tracking Analysis (NTA)

The conditioned media of organoid cultures were harvested after 2 days, centrifuged at 300× *g* for 5 min and at 2000× *g* for 20 min to remove cells, cell debris and apoptotic bodies. After centrifugation, 100 µL supernatant was diluted to 1 mL in PBS and the EV concentration and size distribution were measured on a ZetaView Z-NTA instrument (Particle Metrix, Inning am Ammersee, Germany). The cell positions were scanned at 25 °C with the following camera settings: auto expose, gain: 28.8, offset: 0, shutter: 100, sensitivity: 80. The videos were analyzed with a minimum area of 5, a maximum area of 1000, and minimum brightness of 20 by the ZetaView Analyze software 8.05.10. Cells were cultured under the same conditions (medium volume, time, tissue culture dish format) when EV release from the experimental groups was compared directly. The particle concentration data were normalized to cell numbers.

### 4.4. Functional Experiments with EVs

Conditioned media of organoid cultures were serially centrifuged at 300× *g* for 5 min and 2000× *g* for 20 min at 16 °C to remove cells, cell fragments and apoptotic bodies. Samples were then ultracentrifuged (UC) at 100,000× *g* for 70 min at 4 °C, the EV-containing pellet was resuspended in PBS and UC again. The EV-containing pellets were resuspended in PBS and 10 µL was added to fibroblasts in 24-well plates or 4-well chamber slides (1.2 × 10^4^ cells in 500 µL) in serum-free medium. When the experimental setup required EV quantification, their number was determined with NTA before adding them to the fibroblasts.

In some experiments, sorted CRC cells were cultured with Vybrant Cell-Labeling Solution DiI dye for 10 min (according to the Cell in Suspension protocol of the manufacturer, Thermo Fisher), the cells were washed with DMEM–F12 medium (Gibco) to remove the free dye and were then embedded into Matrigel. The chemically defined medium was changed on day5, EVs were collected for 2 days and they were then isolated with centrifugation and UC. Fibroblasts treated with labelled EVs were fixed with 4% paraformaldehyde (PFA) for 20 min the next day and were then incubated with Phalloidin-iFluor 488 Reagent (Abcam, Cambridge, United Kingdom) for 20 min. After covering samples with ProLong Diamond antifade mountant containing DAPI (Thermo Fisher), images were taken with a Leica TCS SP8 confocal microscope (Wetzlar, Germany) and evaluated with ImageJ software (National Institutes of Health, Bethesda, MD, USA).

### 4.5. Liposome Production

The production and characterization of liposomes have been previously described [22]. The liposomes (produced from phosphocholine and cholesterol with a mean diameter of 105 nm) were added to fibroblasts in 10 µL PBS.

### 4.6. Flow Cytometry and Cell Sorting

Organoids were dissociated into single cells by TryPLE (5–10 min) and were then suspended in FACS buffer (PBS, 1 mM EDTA, 25 mM HEPES, 1% BSA). Cells were labeled with primary antibodies for 20 min on ice and then the secondary antibodies were applied for 20 min at room temperature. 10,000 events were measured with a Cytoflex (Beckman Coulter, Brea, CA, USA) instrument or cell subpopulations were sorted by a cell sorter (Sony SH800S, Sony Biotechnology Inc., Bothell, WA, USA). Sorted cells were centrifuged at 700× *g* for 10 min at 4 °C and 10,000–15,000 cells were embedded into 20 µL Matrigel droplets. Identical cell numbers were used within the same experiment to obtain comparable results.

### 4.7. Detecting EVs by Anti-CD63 and Anti-CD81-Coated Beads

EVs were collected in CRC organoid cultures for 2 days. They were then centrifuged at 300× *g* for 5 min and 2000× *g* for 20 min. EVs were bound to beads coated with anti-CD63 (Thermo Fisher, 10606D) or anti-CD81 (Thermo Fisher, 10616D) that had been blocked with 0.1% BSA (Merck) for 20 min. Six µL and 20 µL of the anti-CD81 or anti-CD63-coated beads were used for 200 µL supernatant, respectively. Beads were incubated overnight at 4 °C, washed with PBS 3-4 times and the bead-bound EVs were detected with FITC-anti-CD81 or PE-anti-CD63 and with flow cytometry (FACSCalibur, BD, East Rutherford, NJ, USA). This method was used only for EV characterization.

### 4.8. Immunocytochemistry

Fibroblasts were fixed in 4% PFA for 20 min and then blocked and permeabilized in blocking buffer (PBS with 0.1% BSA, 5% FBS, and 0.1% Triton X-100). Primary antibodies were applied at 4 °C overnight and then secondary antibodies for 2 h at room temperature (all in blocking buffer). Samples were covered with ProLong Diamond antifade mountant containing DAPI (Thermo Fisher) and images were taken with a Leica TCS SP8 confocal microscope. The antibodies used are listed in Table S1.

### 4.9. Whole-Mount Immunostaining

Patient-derived organoids were cultured in 8-well chamber slides (BD Biosciences, East Rutherford, NJ, USA), fixed in 4% PFA for 30 min, and washed with PBS twice. We used whole-mount blocking buffer (WBB, containing 5% FBS, 0.2% BSA, 0.3% Triton X-100 in PBS) for blocking and permeabilizing the samples for 30 min. Samples were incubated with primary antibodies at 4 °C overnight in WBB, they were washed with PBS, labeled secondary antibodies were applied overnight at 4 °C and the organoids were mounted with ProLong Diamond antifade mountant containing DAPI (Thermo Fisher Scientific). We used a Leica TCS SP8 confocal microscope for imaging and the ImageJ software for analysis and quantification. The antibodies used are listed in Table S1.

### 4.10. Protein Concentration Measurement and Simple Western (WES) Analysis

EVs were pelleted from organoids grown in chemically defined medium by centrifuging (300× *g* for 5 min and 2000× *g* for 20 min) and then ultracentrifugation (100,000× *g*, 70 min at 4 °C). The pellet was washed with PBS, ultracentrifuged again and then resuspended in 19 µL PBS and 1 µL cOmplete™ Protease Inhibitor Cocktail (Roche, Basel, Switzerland). Samples were frozen and thawed several times, the protein concentrations were measured with the Micro BCA Protein Assay Kit (Thermo Fisher Scientific, Waltham, MA, USA) and NanoDrop ND-1000 spectrophotometer (Thermo Scientific). Three µL of the lysates containing 0.5 µg protein were applied to Simple Western analysis WES (ProteinSimple, San Jose, CA, USA) following the manufacturer’s instructions. SM-W004 (molecular size marker), DM-TP01 total protein detection kit, DM-001 anti-rabbit detection kit and PS-ST02EZ-8 EZ Standard Pack 2 were used (ProteinSimple). The primary antibody (TSG101) is listed in Table S1. For evaluation the Compass for SW4.0.1 software (ProteinSimple) was used.

### 4.11. Transmission Electron Microscopy

EVs were pelleted with UC, they were washed with PBS, ultracentrifuged again and then resuspended in 10 µL PBS. Five µL was dried on a 300 mesh grid (Electron Microscopy Sciences, Hatfield, PA, USA). EVs were fixed with 4% glutaraldehyde for 10 min and the grid was washed with water. Samples were incubated in 2% phosphotungstic acid, dried at room temperature and images were taken with a JEM-1011 transmission electron microscope (JEOL, Akishima, Tokio, Japan) equipped with a Morada digital camera (Olympus, Shinjuku, Tokio, Japan) using the iTEM software (Olympus).

### 4.12. RNA Isolation and mRNA Measurements from Cells

Total RNA was isolated with the miRNEasy Micro Kit (Qiagen, Hilden, Germany) according to the manufacturer’s description in 15 µL RNAse-free water. In some experiments, cells were directly sorted into Qiazol lysis buffer (Qiagen). The RNA concentrations were determined with a NanoDrop instrument. For reverse transcription, we used 0.5 µg RNA (in 20 µL final volume) and the SensiFAST cDNA Synthesis Kit (Bioline, Toronto, Canada). Quantitative PCR reactions were carried out with the SensiFAST SYBR Hi-ROX Kit (Bioline) using the SYBR Green method on an ABI 7900HT Fast real-time PCR instrument (384-well format, 5 µL/well volume). Results were evaluated with the following formula: relative expression level = 2^−ΔCt^, where ΔCt = Ct(gene of interest)–Ct(housekeeping gene). The sequences of the primers are summarized in Table S2.

### 4.13. TaqMan Low Density Array (TLDA)

CRC organoid conditioned media (1 mL) were harvested after 48 h, they were centrifuged at 300× *g* for 5 min, 2000× *g* for 20 min, and 12,500× *g* for 20 min. Forty µL anti-CD63 and 20 µL anti-CD81-coated beads were added to the supernatant after centrifugation, the samples were incubated for 16 h at +4 °C and were washed with PBS 3–4 times. EVs bound to beads were then lysed in Qiazol (Qiagen). Total RNA was isolated with the miRNEasy Micro Kit (Qiagen) following the manufacturer’s protocol.

Three µL total RNA was reverse transcribed with Megaplex RT primers, the samples were pre-amplified with Megaplex PreAmp Primers (Thermo Fisher) and the TaqMan™ Array Human MicroRNA A Cards v2.0 (Thermo Fisher) were measured on an ABI 7900HT instrument according to the manufacturer’s protocol and according to [23].

### 4.14. Bioinformatical Analysis

The threshold of TLDA measurement was set to 0.2 and Ct < 35 was regarded as “miRNA present”. The NormFinder algorithm (NormFinder software. Available online: https://moma.dk/normfinder-software (accessed on 5 May 2021)) was used to calculate the expression level stability for each miRNA (Table S3), based on the average pairwise variation between all candidate reference miRNAs [23]. The NormFinder algorithm also takes into consideration both the intra- and inter-group variability. Based on these analyses, miR-19b was selected for normalization, and the ΔCt value was calculated for each miRNA according to the following formula: ΔCt = Ct(miR of interest) − Ct(miR-19b). Before further analysis, we removed miRNAs that were present in at least one of the control samples that contained no cells. Only miRNAs that were detected in all three replicates of the same condition were used. When filtering miRNAs specific for one of the groups, we considered miRNAs present in all the replicates in one condition and absent in all samples of the other condition. For linear discriminant analysis (LDA) with a custom Python script (available from the authors upon request), Ct values were normalized and a Ct value of 35 was used for miRNAs with an undetermined flag to obtain numerical data for statistical tests.

### 4.15. Statistical Analysis

Student’s paired or unpaired *t*-tests, Mann–Whitney U test, Kruskal–Wallis with Dunn post hoc test were used with * *p* < 0.05, ** *p* < 0.01, and *** *p* < 0.005 significance levels. Statistical evaluations were carried out with Microsoft Excel, SPSS version 25, and GraphPad software. Mean + SD or median and 25 percentile values are shown.

## 5. Conclusions

Collectively, we provide evidence that CD44 level marks CRC cell populations not only with a differential proliferation, but also EV secreting capacity. When comparing the effect of EVs from CD44^high^ and CD44^low^ CRC cells on fibroblasts, we found that the different EV number, and not the miRNA cargo was the relevant factor. Thus, our results may significantly contribute to understanding how EVs released from CRC subpopulations act in the tumor microenvironment.

## Data Availability

The data or material of this study are available from the corresponding author upon reasonable request.

## Supplementary Materials

They are available online at https://www.mdpi.com/article/10.3390/ijms23042180/s1.
