# Supplementary material for: CD44 Expression Intensity Marks Colorectal Cancer Cell Subpopulations with Different Extracellular Vesicle Release Capacity
Source: Int J Mol Sci. 2022 Feb 16;23(4):2180. doi: 10.3390/ijms23042180 (PMC8879498; doi:10.3390/ijms23042180)
Supplement: Supplementary file 1 [file ijms-23-02180-s001.zip › Kelemen_Andrea_suppl.pdf]

CD44 expression intensity marks colorectal cancer cell subpopulations with different extracellular vesicle release capacity

Andrea Kelemen, Idan Carmi, Iván Seress, Péter Lőrincz, Tamás Tölgyes, Kristóf Dede, Attila Bursics, Edit I Buzás, Zoltán Wiener*

*corresponding author

Antibody	Source	Clone/Cat No
FITC anti-human CD81	Molecular Probes	A15753
PE anti-human CD63	Sigma-Merck	SAB4700218
anti-KI67	Abcam	Ab16667
anti-human/mouse active caspase-3	Bio-Techne (R&D Systems)	AF835
anti-human CD44-PE	BD Pharmingen	555479
anti-human CD44	Abcam	Ab157107
anti-human CD133	Miltenyi Biotec	130-090-851
anti-human PTK7-APC	Miltenyi Biotec	130-099-660
anti-human PTK7	Miltenyi Biotec	130-091-578
anti-human TSG101	Sigma-Merck	T5701
anti-mouse IgG Alexa 488	Invitrogen/Thermo Fisher	AF21202
anti-mouse IgG Alexa 568	Invitrogen/Thermo Fisher	AF10037
anti-rabbit IgG Alexa 488	Invitrogen/Thermo Fisher	A21206
anti-rabbit IgG Alexa 568	Invitrogen/Thermo Fisher	A11011
anti-rabbit IgG Alexa 750	Invitrogen/Thermo Fisher	A21039

Table S1. Antibodies used in our experiments.

Primer name	Sequence
hCD44_fw	GGCTTTCAATAGCACCTTGC
hCD44_rev	GTTGTTTGCTGCACAGATGG
hPTK7_fw	GCAGTGGCTCTTTGAGGATG
hPTK7_rev	AGGTGAAGTGTGGCTTCCAG
hCD133_fw	GCCTCTGGTGGGGTATTTCT
hCD133_rev	TACCTGGTGATTTGCCACAA
hZEB1_fw	GCTGACTGTGAAGGTGTACC
hZEB1_rev	ACATCCTGCTTCATCTGCCT
hHPRT1_fw	TGAGGATTTGGAAAGGGTGT
hHPRT1_rev	TCCCCTGTTGACTGGTCATT

Table S2. Primers used in RT-qPCR.

miR	Stability value

hsa-miR-19b	0.017
hsa-miR-17	0.018
hsa-miR-320	0.018
hsa-miR-29a	0.019
hsa-miR-20a	0.020
hsa-miR-106a	0.021
hsa-miR-16	0.022
hsa-miR-192	0.022
hsa-miR-484	0.023
hsa-miR-92a	0.024
hsa-miR-148a	0.025
hsa-miR-24	0.026
hsa-miR-222	0.028
hsa-miR-194	0.029
hsa-miR-106b	0.029
hsa-miR-223	0.031
hsa-miR-200c	0.032
hsa-miR-203	0.032
hsa-miR-21	0.033

Table S3. Expression level stability of the miRNAs. Results were generated with the NormFinder (https://moma.dk/normfinder-software) algorithm from all miRNA low-density array samples.

Table S4 (see separate Excel sheet). Ct values from the low-density miRNA arrays.

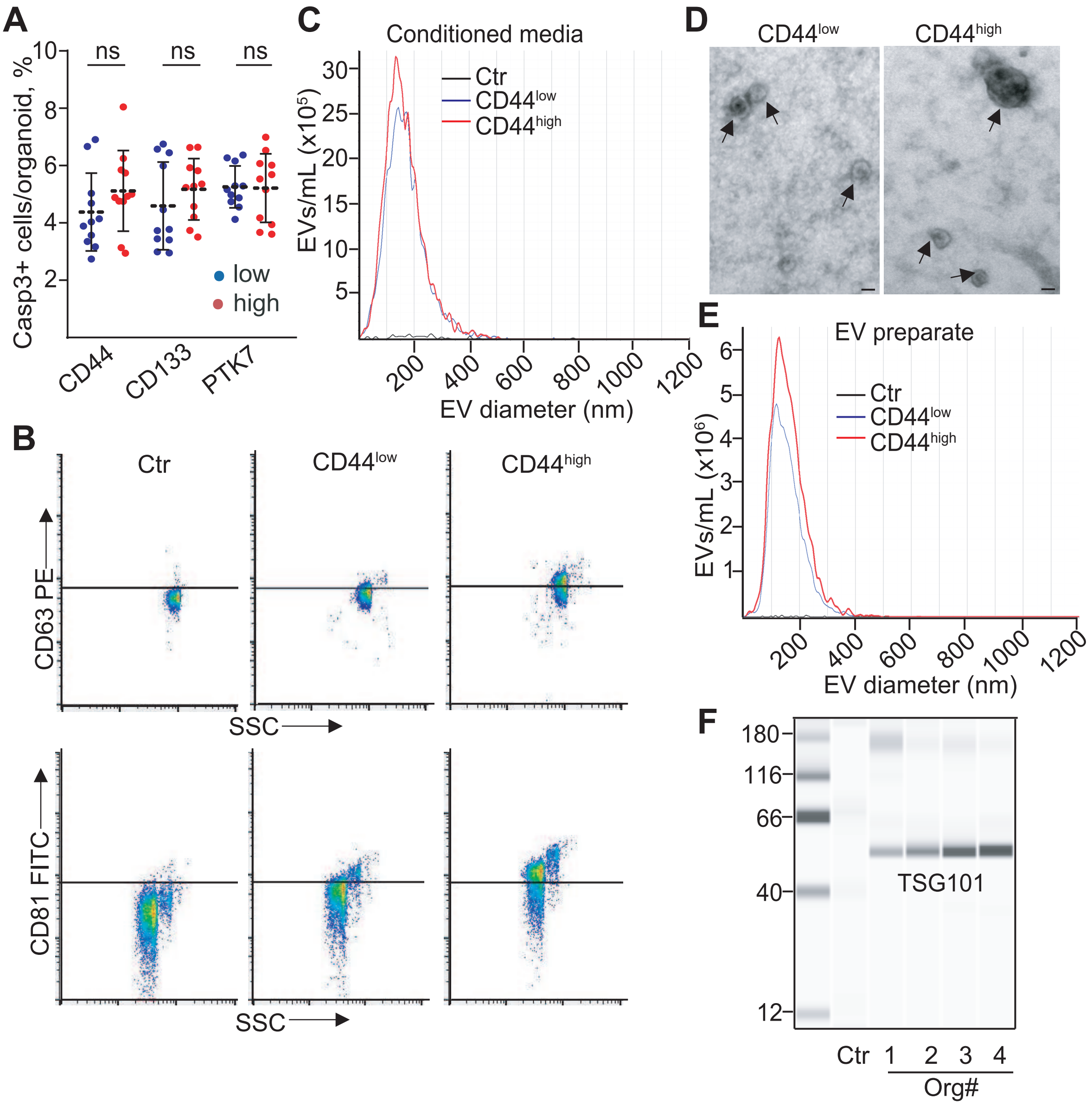

Figure S1. CD44^{high} and CD44^{low} sorted cell-derived organoids release EVs into the supernatant. A) Comparing the percentage of active caspase-3+ apoptotic cells in organoids derived from CD44^{low} and CD44^{high}, CD133^{low} and CD133^{high} and PTK7^{low} and PTK7^{high} cells (quantification of confocal microscopic images. Data were collected from four organoid lines). B) Detecting CD63+ and CD81+ EVs with the bead-based semi-quantitative method and flow cytometry from CD44^{high} and CD44^{low} organoid conditioned media or from medium without organoids (Ctr). Note that beads were coated with anti-CD63 or anti-CD81 antibodies and EVs were detected with anti-CD63 PE or anti-CD81 FITC antibodies, respectively (data from organoid #2). C) Representative Nanoparticle Tracking Analysis (NTA) images from CD44^{high} and CD44^{low} organoid supernatants. Note that Matrigel 3D culture without organoids was used as cell-free control (Ctr). D) Transmission electron microscopic images (TEM) from ultracentrifuged CD44^{high} and CD44^{low} organoid conditioned media at day7 of culturing. E) Representative NTA images from EV preparates of ultracentrifuged CD44^{high} and CD44^{low} organoid supernatants. F) Capillary-based WES immunoblot analysis of ultracentrifuged EV preparates derived from cell-free medium (Ctr) or from the indicated organoid lines. Scale bars: 100 nm (D). Mann-Whitney U-test was used (A).

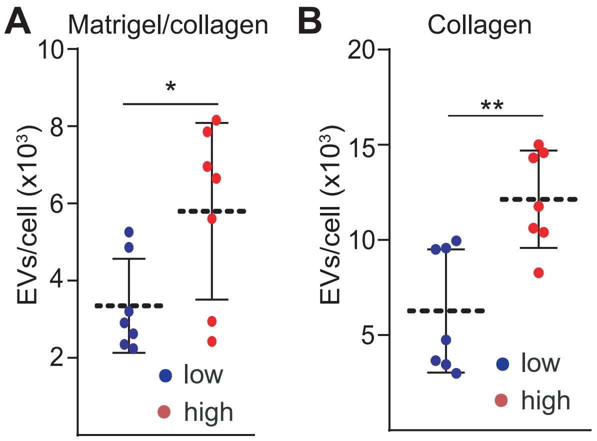

Figure S2. CD44^{high} cells release more EVs compared to CD44^{low} cells in the presence of collagen. too. A-B) EV concentration in organoid supernatants derived from CD44^{high} and CD44^{low} sorted cells on day7 (NTA, 1-2 parallels from 4 organoid lines). Organoids were cultured in Matrigel/collagen I (1:1 ratio. A) or in pure collagen I (B). Mann-Whitney U-test was used (A, B).

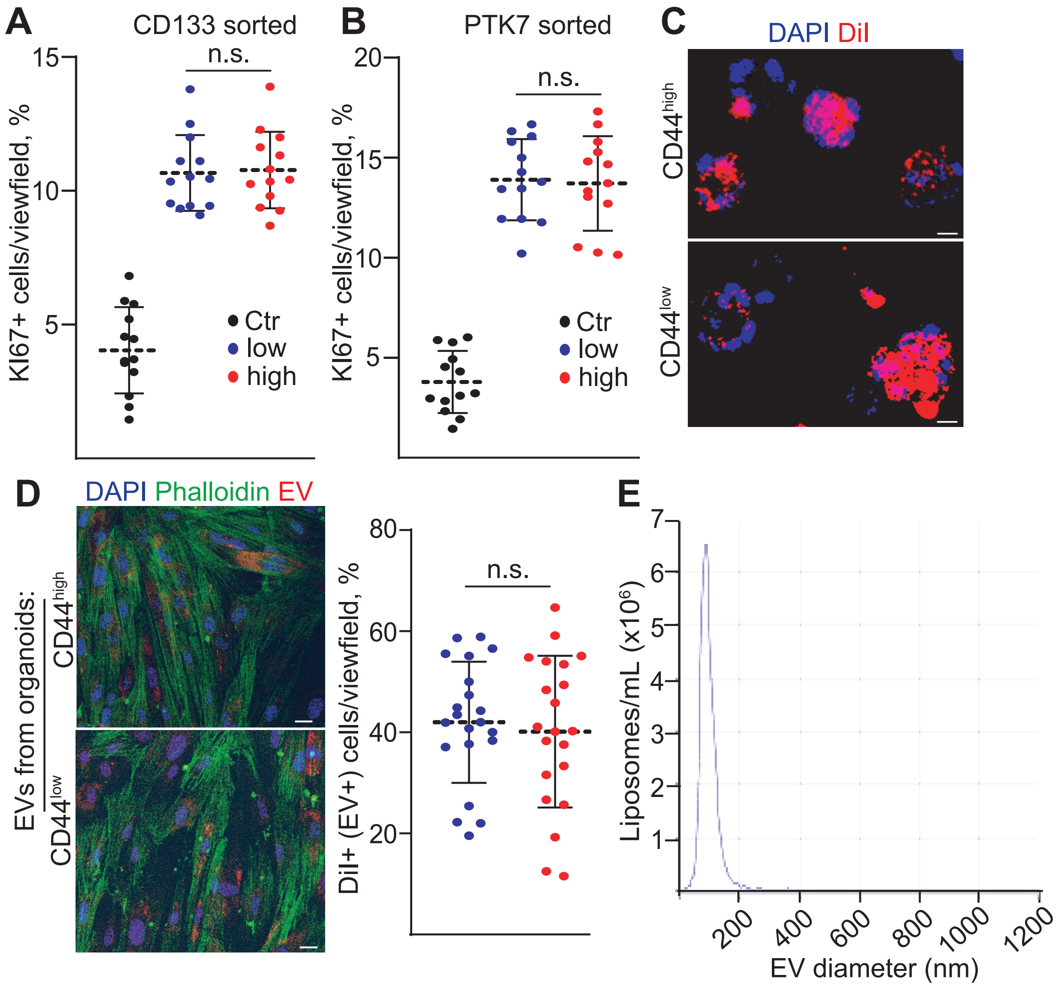

Figure S3. The effect of EVs derived from different CRC cell subpopulations on fibroblasts. A-B) The percentage of KI67+ fibroblast when applying EVs (from 10^6 cells). EVs were collected from the supernatants of CD133^{high} and CD133^{low} (A) or PTK7^{high} and PTK7^{low} (B) cell-derived organoids on day 7. Fibroblasts were treated for 48h (quantification of confocal microscopic images, EVs from four organoid lines were used). C) CD44^{high} or CD44^{low} cell-derived organoids (org #2) labelled with the red fluorescent membrane dye DiI (representative confocal images. D) Colon fibroblasts with fluorescent EV signal. EVs were collected from CD44^{high} or CD44^{low} cell-derived organoids (org #2) labelled with DiI and fibroblasts were treated with the labelled EVs for 24h. Representative confocal microscopic images (left panel) and their quantification. Note that untreated cells showed no red signal (not shown). Phalloidin was used to visualize cells. E) NTA measurement of liposomes used in our studies. Kruskal-Wallis and Dunn tests (A, B) or Mann-Whitney U-test (D) were used with n.s.>0.05. Note that only the interesting comparisons are marked. Scale bars: 10µm (C, D).
